# Countering *in situ* reduction of SnO_2_ during electrochemical CO_2_ conversion *via* oxidative pulsing†

**DOI:** 10.1039/d5ma00272a

**Published:** 2025-07-30

**Authors:** Sven Arnouts, Kevin Van Daele, Nick Daems, Mathias van der Veer, Sara Bals, Tom Breugelmans

**Affiliations:** a Applied Electrochemistry and Catalysis (ELCAT), University of Antwerp 2610 Wilrijk Belgium Tom.Breugelmans@uantwerpen.be; b Electron Microscopy for Materials Science (EMAT) and NANOlight, University of Antwerp 2020 Antwerp Belgium

## Abstract

The application of periodic anodic pulses in CO_2_ electroreduction (p-eCO_2_R) offers a promising route to counteract the inevitable *in situ* reduction of metal oxide catalysts. This study demonstrates the first application of p-eCO_2_R to a catalyst composed solely of a tin (oxide) active phase, using a pomegranate-structured SnO_2_@C nanosphere. Periodic, prolonged anodic pulses (30 s) at 0.2 V *vs.* RHE improved faradaic efficiency towards formate after 6 hours, retaining 78 ± 2% *versus* 71 ± 6% under potentiostatic conditions, suggesting p-eCO_2_R can extend Sn-based catalyst lifetimes for more sustainable CO_2_ conversion.

## Abstract 2

The application of periodic anodic pulses in CO_2_ electroreduction (p-eCO_2_R) offers a promising route to counteract the inevitable *in situ* reduction of metal oxide catalysts.

## Graphical abstract

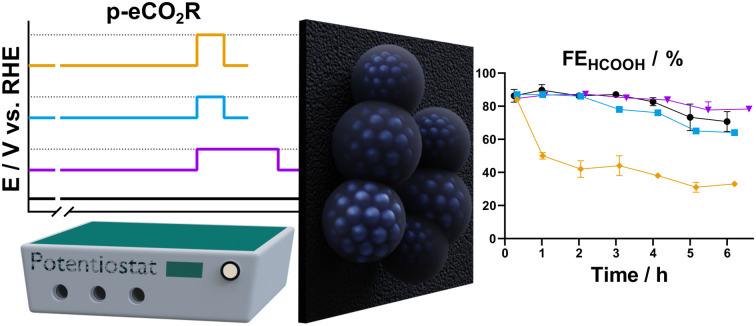

Since the Industrial Revolution, atmospheric CO_2_ levels have risen sharply, driven primarily by human activities such as the extensive use of fossil fuels and widespread deforestation.^1^ This surge in CO_2_ concentration has become a major contributor to global warming, which continues to accelerate at an alarming pace.^2^ While Earth's average temperature has increased by approximately 0.06 °C per decade since 1850, this rate has more than tripled to 0.20 °C per decade since 1982.^3^ To mitigate rising emissions while meeting the growing energy demands of modern industry, the transition to a more circular and sustainable society is increasingly being explored. In this context, the electroreduction of CO_2_ (eCO_2_R) emerges as a promising strategy to combat climate change by recycling carbon dioxide into valuable chemicals and fuels. When coupled with renewable energy sources, eCO_2_R offers a sustainable pathway to produce carbon-neutral commodities, addressing both environmental and energy challenges simultaneously.

Typically, eCO_2_R is conducted under steady-state operating conditions, where either a current or potential is applied to the system and is maintained at a fixed level. While the initial performance of state-of-the-art electrocatalysts meets high industrially relevant standards (*i.e.*, high faradaic efficiency at industrially relevant current densities), prolonged operation typically results in catalyst degradation, impacting the systems efficiency and overall industrial feasibility.^4^ Various studies concerning the stability and degradation of several state-of-the-art eCO_2_R catalysts have reported a multitude of predominant degradation mechanisms, including pulverization, agglomeration and particle detachment, that potentially take place during the eCO_2_R.^5–7^ While these can be mitigated using various techniques, such as employing support materials to immobilize the catalyst,^8,9^ others are intrinsic to eCO_2_R. One such inherent process is the *in situ* reduction of metal oxides, driven by the reductive conditions at the catalyst surface. A promising, nonetheless underexplored, option to counteract this *in situ* reduction, thereby prolonging the lifetime of a catalyst, is the application of pulsed CO_2_ electroreduction (p-eCO_2_R), whereby the steady-state cathodic operation conditions are periodically interrupted by an anodic treatment.^10,11^

Indeed, Li *et al.* established that countering the *in situ* reduction of Cu_*x*_O catalysts and thereby maintaining an optimal Cu_*x*_O/Cu ratio at the catalyst surface is indispensable for eCO_2_R.^12^ Additionally, Engelbrecht *et al.* stated that an anodic bias in a pulse profile can lead to a conservation of the surface structure.^13^ On top of that, the merit of p-eCO_2_R is not limited to the preservation of the catalyst. Kim *et al.* demonstrated that anodic pulsing at high frequencies helps sustain elevated CO_2_ concentrations at the catalyst surface, resulting in an increased faradaic efficiency (FE) towards C_2+_ products. Carefully designing the applied pulse profile thus allows for a variety of physicochemical processes, inherent to heterogeneous electrocatalysis, to be manipulated *in situ*.^14^

Despite the opportunities of p-eCO_2_R to enhance electrocatalytic selectivity and prolong the lifespan of electrocatalysts, its application to Sn-based catalysts remains underexplored. Recently, Woldu *et al.* reported a shift of selectivity for SnS_2_ nanosheets from H_2_ to formate when p-eCO_2_R was applied.^15^ Furthermore, Khiarak *et al.* reported improved eCO_2_R stability of Sn nanoparticles deposited on Ag coated PTFE when a periodic anodic current was applied.^16^ However, to the best of our knowledge, no research has been performed on p-eCO_2_R with Sn (oxide) as the sole active catalytic phase. In our previous research featuring Pom. SnO_2_, the use of a carbon shell has been demonstrated to successfully reduce irreversible morphological degradation, such as segregation/pulverization and agglomeration, which was clearly observed for the pomegranate-structured SnO_2_ electrocatalyst and barely detected for the Pom. SnO_2_@C electrocatalyst.^17^ Counterintuitively, the Pom. SnO_2_@C electrocatalyst which maintained its morphology, displayed a decreasing FE_HCOOH_ over the course of 24 hours. However, selectivity was largely restored after drying, and thereby re-oxidizing, the catalyst over air. Ultimately, this temporary loss of FE_HCOOH_ was attributed to the *in situ* SnO_2_ reduction to metallic Sn.^18^ Combining these observations with the fact that longer anodic pulses (>1 s) have previously been reported to yield surface roughening and morphological changes, as well as the formation of persistent oxides on Cu-based electrocatalysts, it is obvious that p-eCO_2_R could provide a valuable approach to diminish/reverse *in situ* SnO_2_ reduction and thereby prolong Sn-based electrocatalytic stability. Therefore, an exploratory study was performed, investigating the possibility to further enhance the stability of a Pom. SnO_2_@C electrocatalyst. By applying several pulse parameter combinations, an initial idea concerning the effect of a transient potential on the electrochemical performance (*i.e.* selectivity, activity and stability) of the Pom. SnO_2_@C electrocatalyst was acquired.

## Results & discussion

**Fig. 1:**
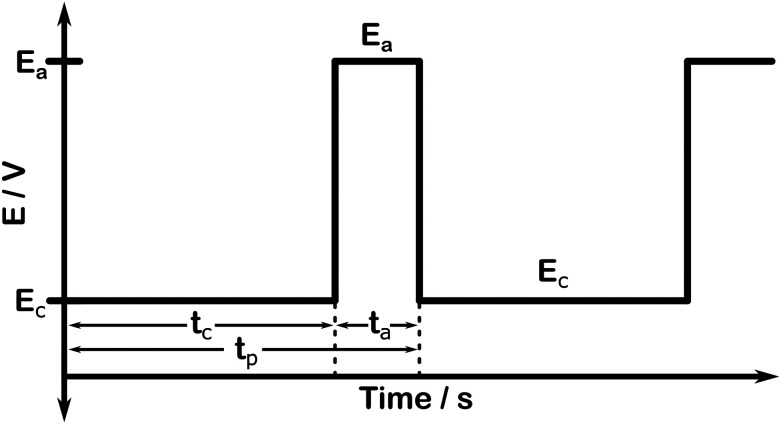
Example of an applied pulse profile for pulsed electrochemical CO_2_ reduction.

Fig. 1 illustrates the key concept behind potential controlled p-eCO_2_R, with *E*_c_ and *E*_a_ the applied potentials during the cathodic and anodic timespan, respectively. The potential is constantly varied between these two values, which depend on the utilized electrocatalyst and the intended goal of the anodic treatment. A square wave pulse profile, as depicted in Fig. 1, is considered the most elementary form of p-eCO_2_R and was utilized here. It should be noted, however, that more complex waveforms, such as triangular, sawtooth, sinusoidal, *etc.*, could be explored once an in-depth understanding of the relationship between the applied pulse and electrochemical performance (electrocatalytic stability) has been attained.^18^

In addition to the potentials, the duration of both the cathodic and anodic pulse (*t*_c_ and *t*_a_, respectively) is variable and determines the total period of one pulse cycle (*t*_p_ = *t*_c_ + *t*_a_) and thus the pulse frequency (*f*_p_ = *t*_p_^−1^). Typically pulses are classified as “short” (<1 s) or “long” (>1 s).^19^ An efficient ratio of *t*_c_ and *t*_a_ exists, *i.e.* the anodic pulse should be long enough to provide the desired effect to the system, but not too long so that the majority of the pulse cycle is utilized to reduce CO_2_ to keep the energy penalty as low as possible.^20,21^

**Fig. 2:**
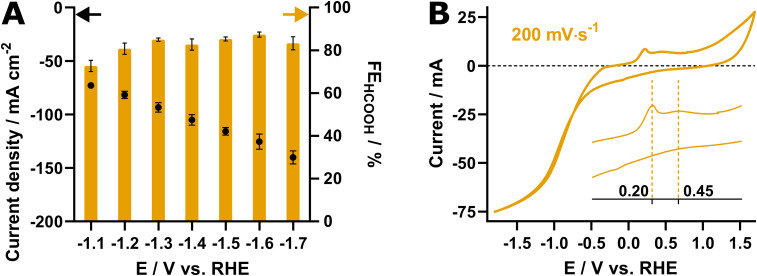
(A) Current densities (black) and faradaic efficiencies (orange) resulting from the potential screening of the Pom. SnO_2_@C catalyst. All measurements were carried out in triplicate; (B) cyclic voltammogram of the SnO_2_@C catalyst recorded at 200 mV s^−1^ under conditions identical to those during eCO_2_R.

While most research in literature is limited to H-cells, our research was conducted in a flow cell using 0.5 M KHCO_3_ as catholyte and a spray-coated gas diffusion electrode (GDE) that was fed with CO_2_ from the backside (more details in the Experimental section). To establish the operating potential for eCO_2_R, a potential screening was conducted to identify the optimal *E*_c_. Therefore, seven different potentials, spanning from −1.1 to −1.7 V *vs.* RHE, were applied and liquid samples were collected and analyzed using HPLC. Fig. 2A displays the results of this potential screening, revealing an excellent performance with selectivities around 80% for all applied potentials. Logically, a large difference in current response was observed, ranging from 73 ± 2 mA cm^−2^ at −1.1 V to 140 ± 6 mA cm^−2^ at −1.7 V *vs.* RHE. Ultimately, −1.4 V *vs.* RHE was chosen as *E*_c_ in the p-eCO_2_R experiments, as the system displayed an excellent FE_HCOOH_ (83 ± 3%) and current density exceeding −100 mA cm^−2^ (−105 ± 5 mA cm^−2^). Although −1.6 V *vs.* RHE yielded a slightly higher FE_HCOOH_ at 87 ± 1%, applying such a strong negative potential would drastically increase the degradation rate beyond the previously reported and possibly introduce additional complications, out of scope of combating *in situ* SnO_2_ reduction in SnO_2_-based CO_2_ electroreduction catalysts.

The *E*_a_ was determined by performing cyclic voltammetry (Fig. 2B) at 200 mV s^−1^ under eCO_2_R conditions. The voltammogram revealed two peaks, one near 0.2 V *vs.* RHE and one near 0.45 V *vs.* RHE, respectively, which are attributed to the oxidation of *in situ* reduced Sn^0^ to Sn^2+^ (0.2 V) and Sn^4+^ (0.45 V). As metastable Sn^2+^ oxyhydroxide was established as the active site for the selective eCO_2_R towards formic acid by Baruch *et al.*, 0.2 V *vs.* RHE was chosen as *E*_a_ in order to steer the re-oxidation towards Sn^2+^.^22^ Gupta *et al.* determined that for a typical boundary layer with a thickness of approximately 100 μm, a *t*_a_ of 5–10 s is required for the effect of the anodic treatment to reach the catalyst surface.^19^ Since we aim to surpass this and go for re-oxidation of the catalyst (surface), an initial *t*_a_ of 10 seconds was used in this work. The *t*_c_ was set to 300 s, resulting in a total pulse period of 310 s and limiting the time lost for eCO_2_R to 3%.

To confirm that effective re-oxidation of the catalyst (surface) is possible under this regime, *in situ* Raman spectroscopy was performed on GDEs coated with SnO_2_@C pomegranates. Since the setup consists of a one-compartment 3-electrode cell equipped with an Ag pseudo-reference electrode, the potentials established *ex situ* are not directly transferrable. Rather, a second cyclic voltammetry experiment was executed to determine the relevant potentials for oxidation and reduction (Fig. S1, ESI†). To accord with the p-eCO_2_R experiments, the potential at the oxidation peak of Sn^0^ to Sn^2+^, here located at −0.1 V *vs.* Ag, was chosen as *E*_a_. The *E*_c_ was set 1.6 V more negative, at −1.7 V *vs.* Ag, thereby maintaining the same potential difference between *E*_a_ and *E*_c_ as that was used in the *ex situ* experiments. Furthermore, a reference Raman spectrum was recorded utilizing commercially available SnO_2_ nanoparticles, confirming that the peak in our region of interest (ROI) originated from SnO_2_ (Fig. S2, ESI†).

**Fig. 3:**
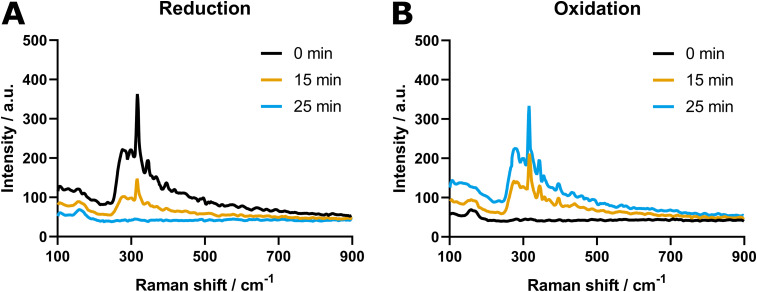
Region of interest of the *in situ* Raman spectra of SnO_2_@C pomegranates under (A) reductive and (B) oxidative conditions. Both images show the spectra recorded at the start, after 15 and after 25 minutes, respectively.

Fig. 3 shows the ROI of spectra resulting from the *in situ* Raman experiments. Primarily, A benchmark spectrum was recorded showing a peak at a Raman shift of 316 cm^−1^ with an intensity of 390 a.u. (Fig. 3A). After applying the *E*_c_ (−1.7 V *vs.* Ag), the peak intensity gradually reduced over the course of time until it completely disappeared after 25 min of reduction. Subsequently, the *E*_a_ (−0.1 V *vs.* Ag) was applied to the system, resulting in the reappearance of the peak at 316 cm^−1^ (Fig. 3B). However, while a gradual decrease in peak intensity was observed during reduction, the increase in the oxidation phase emerged more stepwise. After 25 minutes, the peak reached its maximum intensity at 334 a.u., which equals 86% of its original value before the start of the experiment. To ensure this loss in intensity did not result from exposure to the laser beam, the evolution of peak intensity was studied during a control experiment at open cell potential, which demonstrated no degradation. The reduction in peak intensity is thus assumed to be the result of incomplete reoxidation of the SnO_2_, which is most probably limited to the atomic layers located at the surface of the catalyst.

**Fig. 4:**
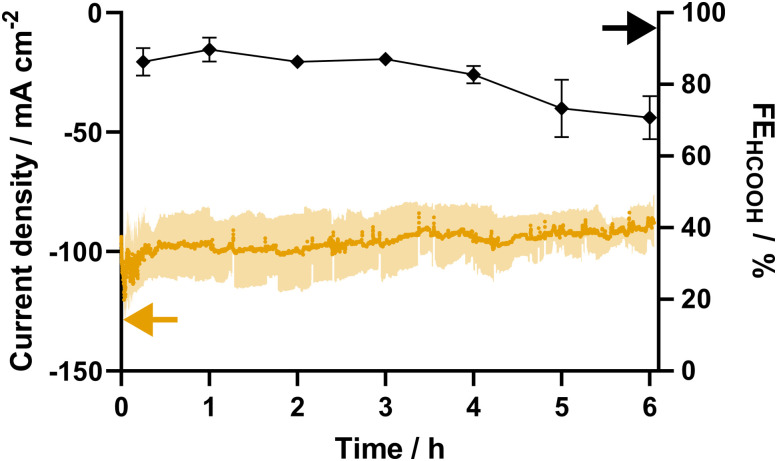
Current response (orange) and faradaic efficiency (black) towards formate for eCO_2_R at −2.1 V *vs.* Ag/AgCl using Pom. SnO_2_@C. The experiment was carried out in threefold.

In order to validate the *E*_c_ and establish a baseline stability, a 6-hour steady-state potentiostatic eCO_2_R experiment was performed in threefold at −1.4 V *vs.* RHE (Fig. 4). Starting at an average current density of −100 ± 14 mA cm^−2^ and a FE_HCOOH_ of 86 ± 4%, the catalyst exhibits a comparable activity and selectivity to that observed during the current screening. After 4 hours, a first decrease in FE is noticeable, which continues, resulting in a FE_HCOOH_ of 71 ± 6% after 6 h of potentiostatic electrolysis. These results are in line with a 6 hour galvanostatic experiment carried out at a current density of −100 mA cm^−2^, which results in a FE_HCOOH_ of 74% after 6 hours (Fig. S2, ESI†).

Given the clear degradation of the catalyst observed within 6 h of eCO_2_R, during which a loss in selectivity of 15% is observed, the duration of p-eCO_2_R experiments was limited to the same timeframe. As mentioned before, the initial parameters were determined by current screening, cyclic voltammetry, and after literature review and were set to 300 s at −1.4 V *vs.* RHE and 10 s at 0.2 V *vs.* RHE for the cathodic and anodic pulse, respectively. Consequently, the Pom. SnO_2_@C catalyst was subjected to 72 pulse cycles, which equals a total *t*_c_ of 6 h. Samples were collected at 15 minutes and after each hour (12 cycles) during the last 120 s of *t*_c_. Experiments were terminated following the anodic segment of the final pulse.

**Fig. 5:**
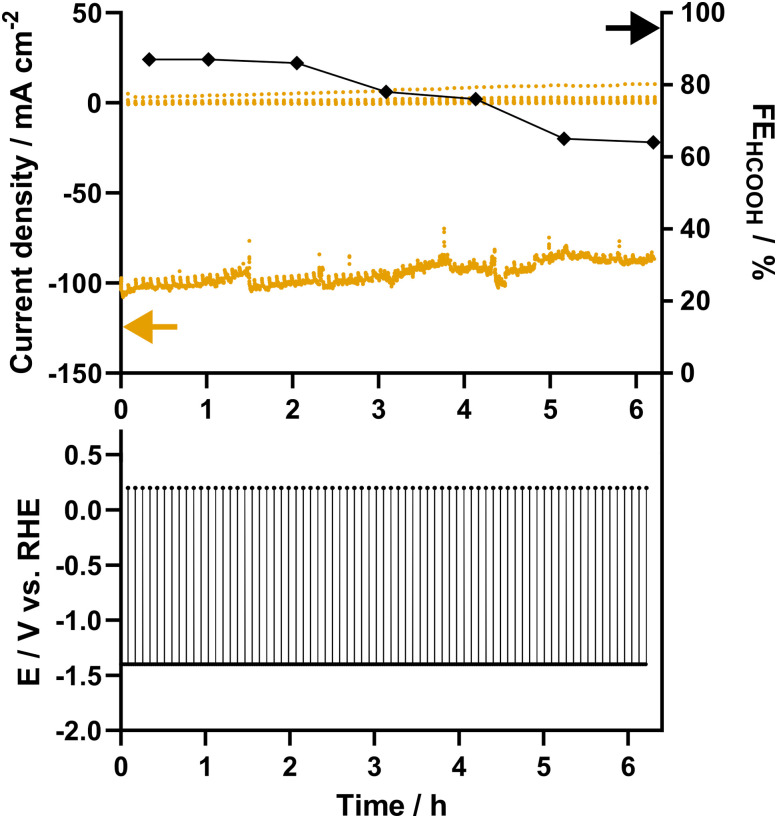
Current density (orange) and faradaic efficiency (black) towards formate and pulse profile resulting from a p-eCO_2_R experiment with *t*_c_ = 300 s, *E*_c_ = −1.4 V *vs.* RHE, *t*_a_ = 10 s and *E*_a_ = 0.2 V *vs.* RHE (p-0.20–10).

After 6 h, a similar current response and decrease in FE_HCOOH_ was observed (Fig. 5) as compared to the steady-state benchmark (Fig. 4). Clearly, the *in situ* reduction of the SnO_2_@C catalyst was insufficiently countered using the aforementioned pulse parameters. On the other hand, the application of a pulsed regime posed no adverse effects on the electrochemical performance. It was therefore hypothesized that either prolonging the *t*_a_ or changing the *E*_a_ towards a more oxidative potential could improve catalyst stability and the *in situ* reoxidation.

Two additional sets of experiments were carried out to combat the *in situ* SnO_2_ reduction and to acquire insight into the effects of a transient potential on SnO_2_-based electrocatalysts. In one, the *t*_a_ was kept at 10 s, while the *E*_a_ was raised to 0.45 V *vs.* RHE, in order to accord with the second oxidation peak of Sn^2+^ to Sn^4+^. In the second, the *E*_a_ was kept at 0.20 V *vs.* RHE, while the *t*_a_ was prolonged to 30 s. The amount of pulses was kept at 72, resulting in an equal total *t*_c_ compared to previous experiments of 6 hours. The resulting current densities and faradaic efficiencies are given in Fig. S4 and S5 (ESI†). From here on, the sets of p-eCO_2_R experiments are named p-0.20–10, p-0.20–30 and p-0.45–10 for the experiments with *E*_a_ = 0.20 V *vs.* RHE for *t*_a_ = 10 s, *E*_a_ = 0.20 *vs.* RHE for *t*_a_ = 30 s, and *E*_a_ = 0.45 V *vs.* RHE for 10 s, respectively.

**Fig. 6:**
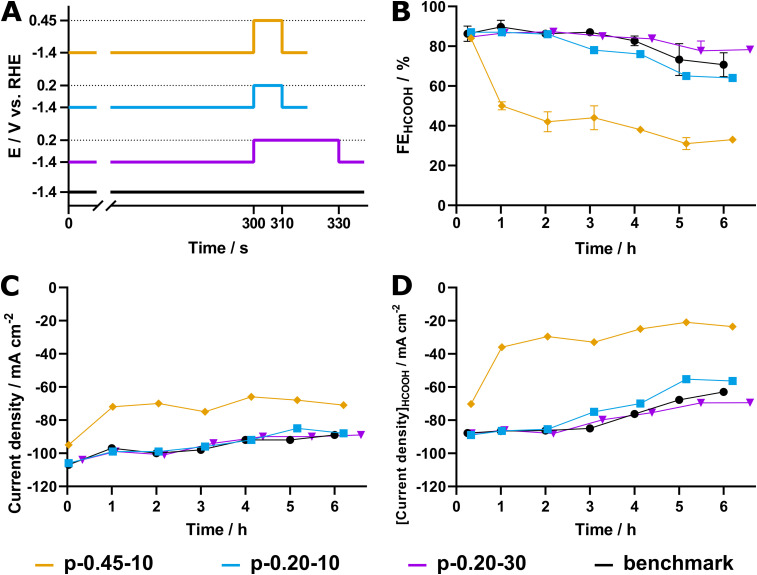
(A) Pulse profiles, (B) faradaic efficiencies towards formate, (C) current densities and (D) partial current densities formate production for 6 h (p)-eCO_2_R experiments labelled p-0.45–10 (orange), p-0.20–10 (blue), p-0.20–30 (magenta) and the potentiostatic benchmark experiment (black).

The resulting pulse profiles for all sets of experiments are visualized in Fig. 6A. The results of the p-eCO_2_R experiments are shown in Fig. 6B–D and compared to those of the steady-state regime. Evidently, p-0.45–10 leads to rapid decay of the faradaic efficiency as well as the current density. While the current response starts at approximately −100 mA cm^−2^, it decreases with each cycle during the first hour of cathodic operation (12 cycles), to finally stabilize at a value around −70 mA cm^−2^. Simultaneously, the FE_HCOOH_ quickly decreased from an initial 84% to a mere 50% after just one hour and only 33% after 72 cycles (6 hours). It is clear that the application of a more oxidative potential has a detrimental effect on the Pom. SnO_2_@C catalyst, resulting in accelerated degradation of its activity.

Contrarily, elongation of the pulse time from 10 to 30 s (*p*-0.20–30) results in a positive effect on the catalyst's stability. The FE_HCOOH_ is measured at 78 ± 2% after 72 cycles of p-eCO_2_R, a decrease of only 6%, which is not only an improvement compared to the 15% loss measured with p-0.20–10, but even to the steady-state potentiostatic conditions, which displayed an FE_HCOOH_ of 71 ± 6% after 6 hours. Additionally, the current density of p-0.20–30 is on par with both p-0.20–10 and the steady-state experiments. As a result, p-0.20–30 outperforms the potentiostatic experiments as well as p-0.20–10 with a specific current density of −70 mA cm^−2^ compared to −63 mA cm^−2^ and −56 mA cm^−2^, respectively (Fig. 6D). Finally, owing to the combination of a decrease in both total current density and FE_HCOOH_, the p-0.45–10 experiment only retains a partial current density towards formate of −24 mA cm^−2^ after 6 hours of cathodic operation.

**Fig. 7:**
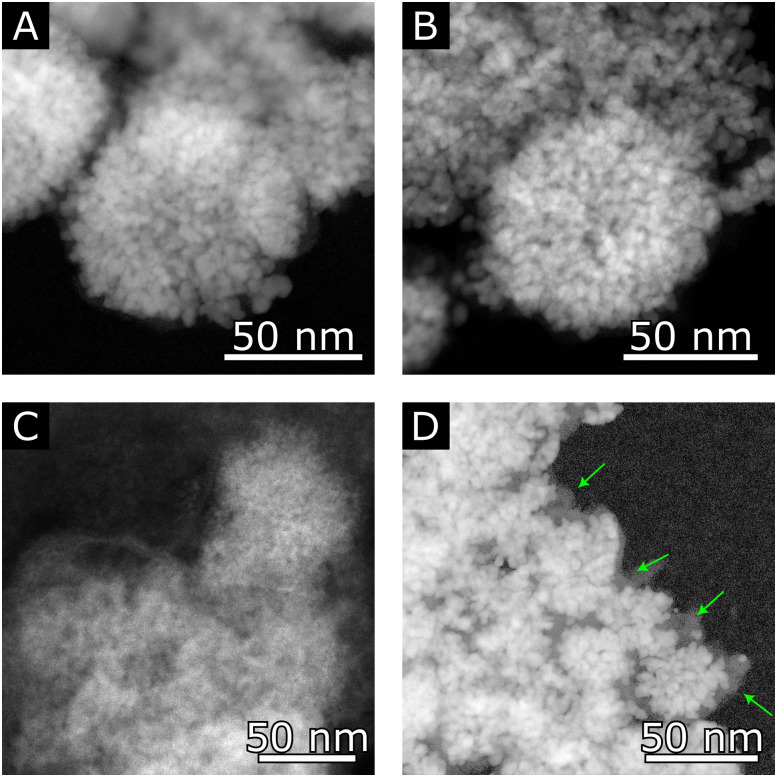
HAADF-STEM images of the SnO_2_@C catalyst in (A) it's pristine form; (B) and (D) after 6 h of p-eCO_2_R under the p-0.20–30 regime; (C) after 6 h of p-eCO_2_R under the p-0.45–10 regime. In (D), the carbon shell, which remained intact after 6 h of p-0.20–30, is indicated with green arrows.

The reduced activity of the SnO_2_@C catalyst subjected to an *E*_a_ of 0.45 V *vs.* RHE (p-0.45–10) can be attributed to damaging changes in its morphology. Fig. 7 shows representative high angle annular dark field scanning transmission electron microscopy (HAADF-STEM) images taken from the SnO_2_@C catalyst, both pristine (Fig. 7A) and after p-eCO_2_R (Fig. 7B–D). While the catalyst originating from p-0.20–30 shows perfect structure retention with intact pomegranate structures (Fig. 7B), Fig. 7C shows a large structure resulting from pulverization and subsequent agglomeration of the original pomegranate structures after p-0.45–10. (Complete agglomerates are shown in Fig. S8, ESI†) Our previous research already underlined the importance of the carbon shell in the retention of the pomegranate structure.^17^ Indeed, it is observed that the pomegranates subjected to an *E*_a_ = 0.20 V *vs.* RHE show an intact carbon shell (Fig. 7D), contrary to the particles after p-eCO_2_R with 0.45 V *vs.* RHE, where no carbon shell remains. Evidently, the higher oxidative potential (*E*_a_) leads to carbon oxidation and successive pulverization/agglomeration of the pomegranate nanoparticles. The rapid decrease in current density and faradaic efficiency observed during the first hour of p-0.45–10 suggest this process happens during the first oxidative pulses, after which the system stabilizes and the pulverization/agglomeration slows down the decrease in FE_HCOOH_ and current density, as previously reported.^17^

It is clear that retention of the original catalyst structure is an important condition to prolong the catalyst lifetime and that the potential applied during the oxidative pulse (*E*_a_), together with its duration (*t*_a_), play a pivotal role in this respect. From our research, it was determined that an oxidative pulse of 30 s at 0.20 V *vs.* RHE, which accords with the oxidation of Sn^0^ to Sn^2+^, is able to effectively re-oxidize the SnO_2_@C pomegranate catalyst without altering its morphology, leading to improved FE_HCOOH_ over time compared to a potentiostatic regime. Combined with a cathodic pulse time of 300 s, the time lost for eCO_2_R is kept at 10%, which is more than compensated by the higher selectivity towards formate and, presumably, a longer lifetime of the catalyst, thus avoiding downtime and costs through catalyst substitution. Future research should, therefore, include experiments that aim for longer duration to further assess the stability and effectiveness of the p-eCO_2_R strategy, as well as investigate its applicability to other Sn-based catalyst.

## Conclusions

In conclusion, an exploratory study towards the merits of applying an *in situ* oxidative pulse on the stability of Pom. SnO_2_@C structures for the eCO_2_R was performed. After a potentiostatic screening and cyclic voltammetry, the cathodic and anodic potential were set to −1.4 V and 0.2 V *vs.* RHE, respectively. *In situ* Raman experiments confirmed that the *in situ* reduced electrocatalyst could be successfully reoxidized under this regime. During p-eCO_2_R experiments, the application of a 10-second anodic pulse following 300 seconds of cathodic operation proved insufficient to counteract the *in situ* reduction, with no observable improvement in faradaic efficiency compared to potentiostatic benchmark experiments. While imposing a more oxidative anodic potential (0.45 V *vs.* RHE) was detrimental for the morphology of the SnO_2_@C catalyst, prolonging the anodic pulse time to 30 seconds proved effective, resulting in an FE_HCOOH_ of 78 ± 2% after 6 hours of cathodic operation, compared to 71 ± 6% for the benchmark experiment at nearly identical current densities. HAADF-STEM imaging conducted after the experiments revealed excellent retention of the SnO_2_@C pomegranate morphology. Despite the limited time span of 6 h, these preliminary experiments demonstrate the merit of p-eCO_2_R, revealing a significant increase in stability for the Pom. SnO_2_ structures. Further optimization of the pulse parameters, along with a comprehensive (*in situ*) study of the catalyst's oxidation state during cycling, should form the scope of future work, to unravel and achieve the full potential of p-eCO_2_R for SnO_2_ and, by extension, other metal oxide electrocatalysts (*e.g.* Cu and Bi) which are inherently prone to *in situ* reduction.

## Author contributions

S. A. and K. V. D. performed the electrochemical measurements and prepared the manuscript. S. A. operated the TEM and interpreted the data. M. V. D. V. performed the *in situ* Raman experiments. N. D. supervised the project. S. B. and T. B. reviewed the manuscript and funded the project. All authors read and approved the manuscript.

## Conflicts of interest

There are no conflicts to declare.

## Supplementary Material

MA-006-D5MA00272A-s001

## Data Availability

Supporting data are given in the uploaded ESI† of the article. The (processed) data will be published and made openly and freely available through deposition in the Zenodo repository of the University of Antwerp and Applied Electrochemistry and Catalysis (ELCAT) Research Group. https://zenodo.org/communities/uantwerp-elcat/.
