# Supplementary material for: Countering in situ reduction of SnO2 during electrochemical CO2 conversion via oxidative pulsing
Source: Mater Adv. 2025 Jul 30;6(17):5857–63. doi: 10.1039/d5ma00272a (PMC12309465; doi:10.1039/d5ma00272a)
Supplement: MA-006-D5MA00272A-s001 [file MA-006-D5MA00272A-s001.pdf]

Supplementary Information (SI) for Materials Advances.
This journal is © The Royal Society of Chemistry 2025

Supporting Information to

Countering *in situ* reduction of SnO_2 during electrochemical CO_2 conversion via oxidative pulsing

Sven Arnouts[a,b†], Kevin Van Daele[a†], Nick Daems[a], Mathias van der Veer[a], Sara Bals[b], Tom Breugelmans[a*]

[a] *Applied Electrochemistry and Catalysis (ELCAT), University of Antwerp, 2610 Wilrijk, Belgium.*

[b] *Electron Microscopy for Materials Science (EMAT) and NANOlight, University of Antwerp, 2020 Antwerp, Belgium.*

† These authors contributed equally to this work.

* Corresponding author

E-mail address: Tom.Breugelmans@uantwerpen.be

Methods

1.1 Chemicals

The following chemicals were used as received, without any further purification: d-glucose (anhydrous, biotechnology grade, VWR Life Science), D520 NAFION® solution (Ion Power), ethanol (99.8%, abs. p., Chem-Lab), potassium hydrogen carbonate (99.5+%, v.p., Chem-Lab), potassium hydroxide (85+%, pellets a.r., Chem-Lab), propanol-2 (99.8+%, iso-propanol a.r., Chem-Lab), sodium tin(IV) oxide trihydrate (98%, Alfa Aesar) and tin(IV) oxide (<100 nm, Sigma-Aldrich).

1.2 Catalyst synthesis

The SnO_2@C pomegranate nanoparticles were synthesized through a method adapted from Wen *et al*.[1] Primarily, 20 mmol sodium stannate ($Na_2SnO_3{\cdot}3H_2O$) was dissolved in 100 mL of a 1 M aqueous glucose solution. The solution was sonicated for 1 hour and then transferred into two Teflon-lined stainless steel autoclaves which were placed in an oven at 180 °C. After 4 hours, the autoclaves were rapidly cooled down to room temperature and the precipitates were collected via centrifugation and subsequently washed three times with deionized water and ethanol. The precipitates were dried overnight at 100 °C and subdued to a final heat treatment at 550 °C (2 °C min^{-1}) for 4 hours under argon atmosphere, after which the pomegranate SnO_2@C nanostructures were obtained.

1.3 Catalyst film preparation

The as-synthesized pomegranate-structured SnO_2@C electrodes were prepared by spray coating a Sigracet® 39BB GDE with a catalyst-containing ink. Hereto, 75 mg SnO_2@C pomegranate nanoparticles were dispersed together with 0.3750 g of a 5 wt% Nafion® solution in 10 mL of a 1:1 mixture of isopropanol and Milli-Q (18.2 MΩ cm at 25 °C). This suspension was slowly and uniformly spray coated onto a GDE of 25 cm^2, resulting in a catalyst loading of approximately 1.5 mg cm^{-2}. Hereafter, the GDE was divided into 6 individual pieces of 3 cm^2, which were used as cathode in the reactor with a geometrical active surface area of 1 cm^2.

1.4 Electrochemical measurements

1.4.1 Cyclic voltammetry

Cyclic voltammetry experiments were carried out stepwise, using a wider potential range for each sequential step, starting from [1.7 -0.3] to [1.7 -0.8], [1.7 -1.3] and finally [1.7 -1.8] V vs. RHE. Each potential window was scanned for a total of 5 cycles at a scan rate of 200 mV s^{-1}.

1.4.2 p-eCO_2R

p-eCO_2R experiments were conducted in the flow-by electrolyzer (**Figure S6**). The catholyte, 0.5M $KHCO_3$, was pumped single pass at a flow rate of 2 mL min^{-1}, while the anolyte, 2M KOH, was recycled at a flow rate of 2 mL min^{-1} over a Ni foam anode. The compartments were separated by a Nafion® 117 membrane. The reference electrode was a 1 mm leak-tight Ag/AgCl electrode. Liquid samples were collected in order to determine the FE towards formate by means of HPLC. The reported data was reproduced three times, with the exception of the 6 h galvanostatic experiment. The average value is reported for all FE_{HCOOH} and current densities.

1.5 HAADF-STEM

The sample of the pristine pomegranate SnO_2@C catalyst was prepared by dispersing the catalyst particles in EtOH before drop-casting on a TEM grid. The degraded samples were prepared by scraping the SnO_2@C catalysts from the GDEs using a scalpel. Hereafter, the catalyst particles were dispersed in EtOH and drop-casted on a TEM grid.

High-angle annular dark field scanning transmission electron microscopy (HAADF-STEM) images were acquired using an aberration-corrected, monochromated Thermo Fisher Scientific Titan 'cubed' electron microscope operated at 300 kV.

1.6 *in situ* RAMAN

In situ Raman measurements were performed in a specifically designed flow cell from Ventacon UK (**Figure S7**). A GDE of 10 x 3 mm was used as working electrode. A gold finger provided electrical contact. A silver rod acted as a pseudo-reference electrode while a Pt ring served as counter electrode. The electrolyte was 0.5 M $KHCO_3$ with a flow rate of 0.05 mL min^{-1}. The Raman spectra were recorded using a red laser with 785 nm wavelength.

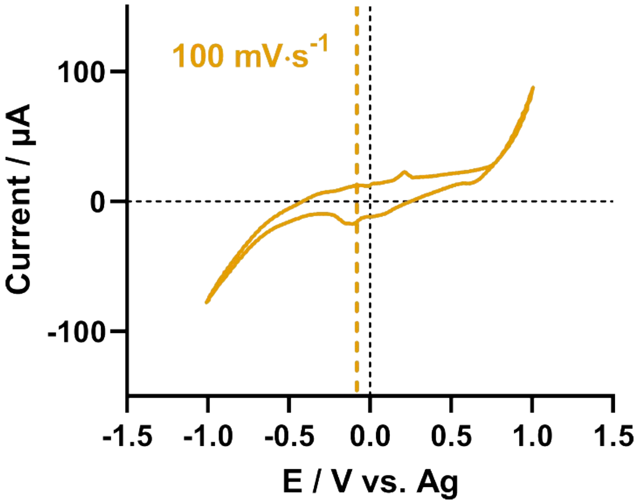

Figure S1 Cyclic voltammogram of the SnO_2@C catalyst recorded in the in situ Raman cell at 100 mV s^{-1} with 0.5 M $KHCO_3$ as electrolyte. The image shows the 5th cycle.

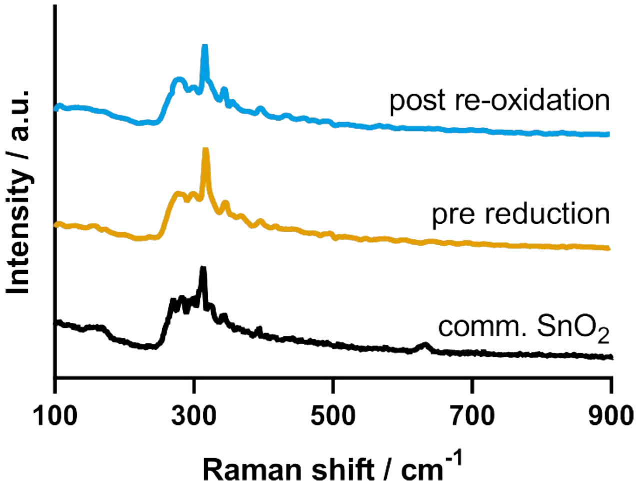

Figure S2 Comparison of the Raman spectra of commercial SnO_2 nanoparticles (black), and the SnO_2@C pomegranates before reduction (orange) and after re-oxidation (blue) in the in situ Raman cell.

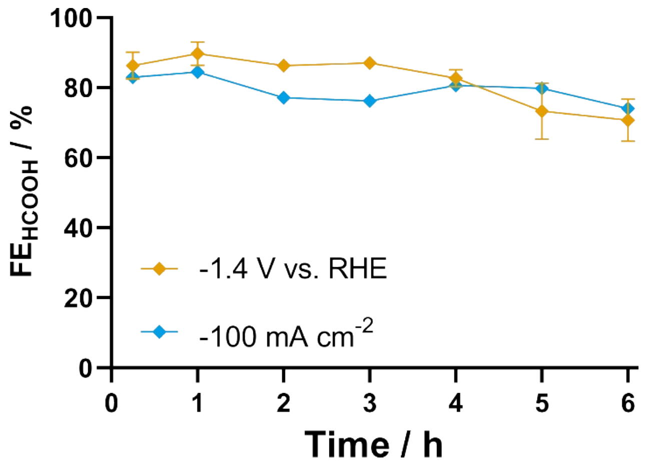

Figure S3 Faradaic efficiencies towards formate for eCO_2R using SnO_2@C pomegranate structures under both potentiostatic and galvanostatic control.

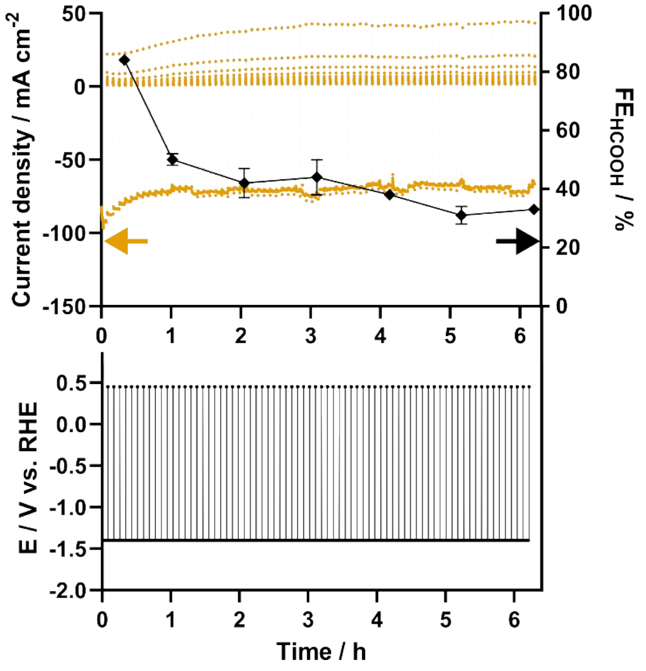

Figure S4 Current density (orange) and faradaic efficiency (black) towards formate and pulse profile resulting from a p-eCO_2R experiment with t_c = 300 s, E_c = -1.4 V vs. RHE, t_a = 10 s and E_a = 0.45 V vs. RHE (p-0.45-10).

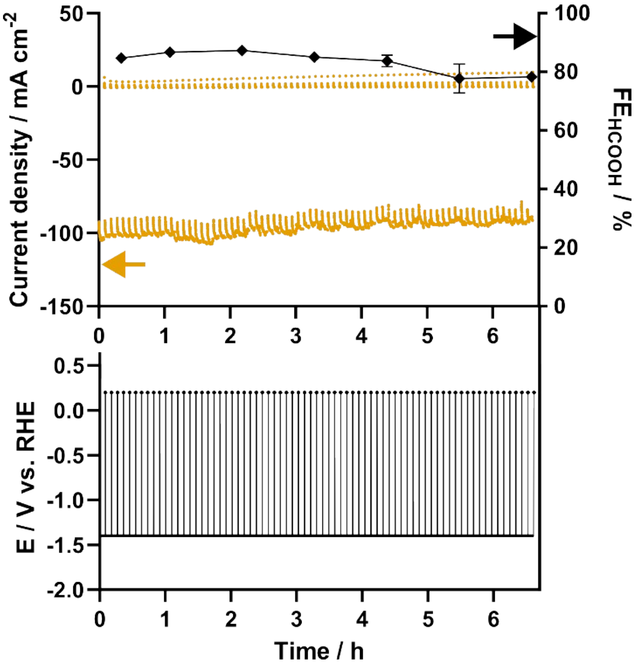

Figure S5 Current density (orange) and faradaic efficiency (black) towards formate and pulse profile resulting from a p-eCO_2R experiment with t_c = 300 s, E_c = -1.4 V vs. RHE, t_a = 30 s and E_a = 0.20 V vs. RHE (p-0.20-30).

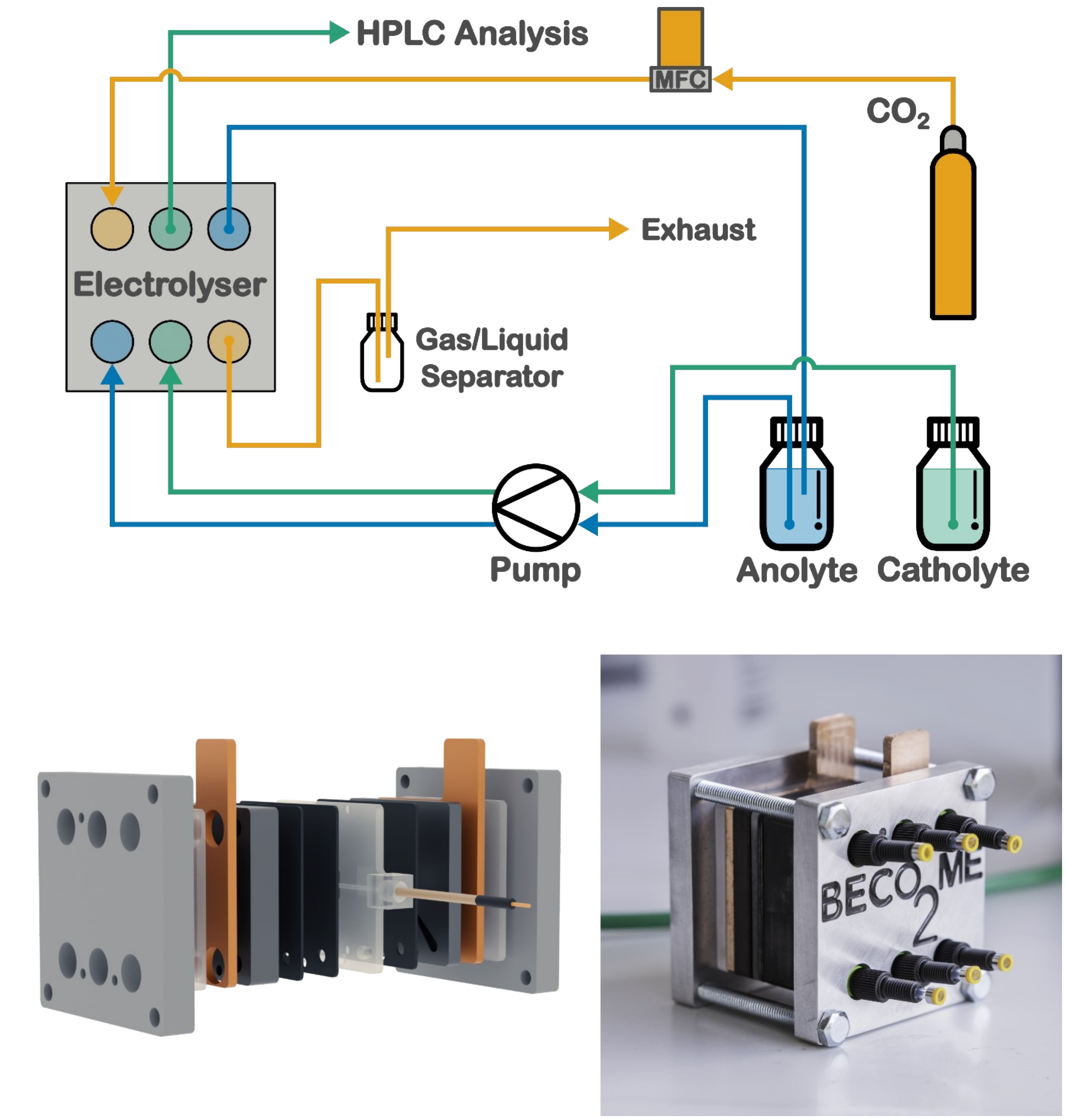

Figure S6 (p)-eCO_2R electrolyzer setup, reproduced with permission from Van Daele et al.[2]

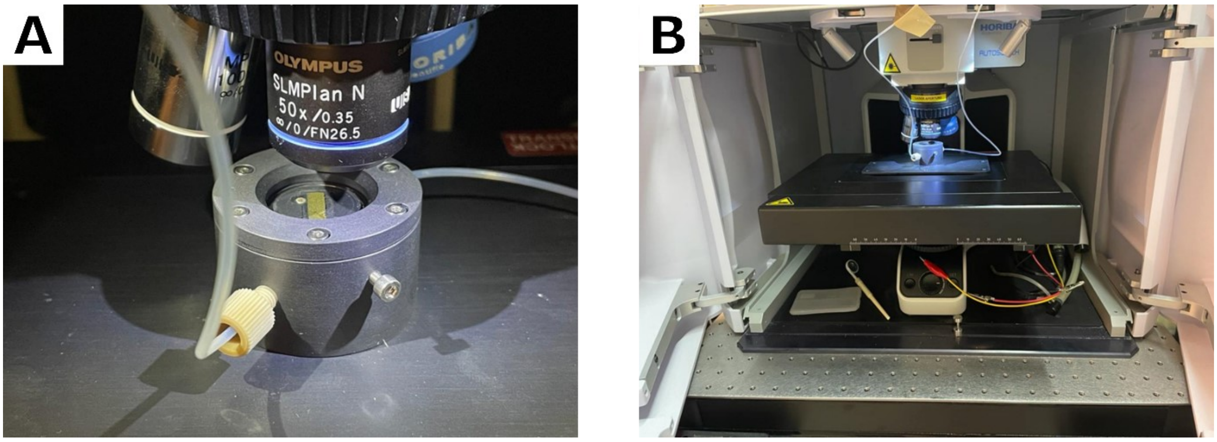

Figure S7 In situ Raman setup with a zoom of the flow cell in (a) and overview in (b).

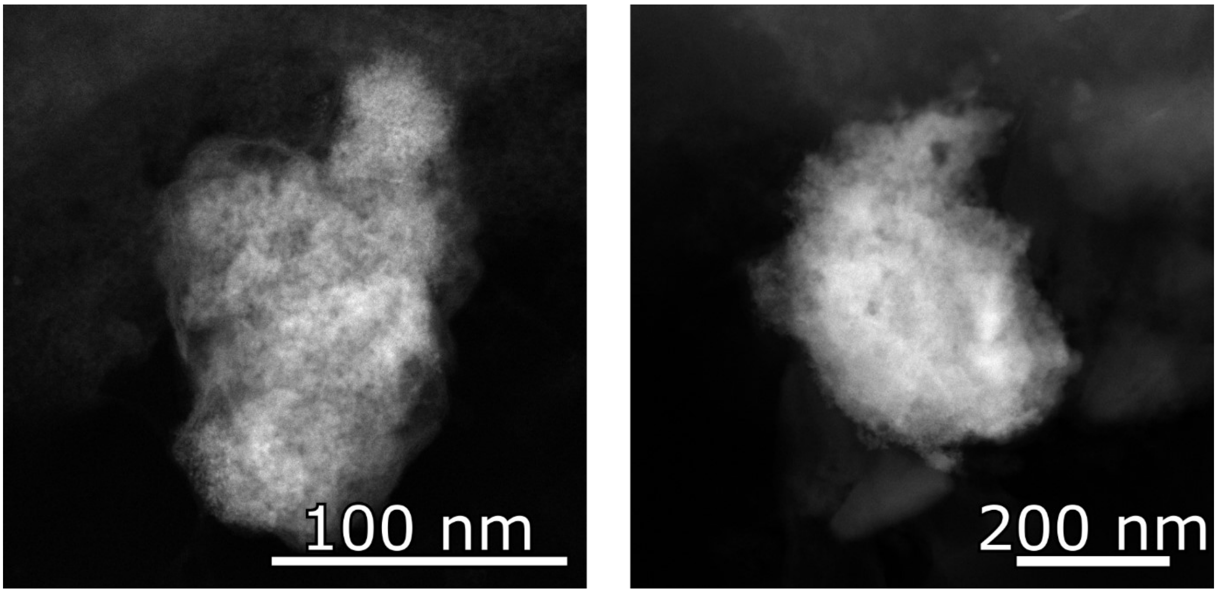

Figure S8 Images showing agglomerated structures stemming from SnO_2@C pomegranate nanoparticles after p-0.45-10.

References

1 W. Wen, M. Zou, Q. Feng, J. Li, L. Guan, H. Lai and Z. Huang, Cu particles decorated pomegranate-structured SnO_2@C composites as anode for lithium ion batteries with enhanced performance, *Electrochimica Acta*, 2015, **182**, 272–279.

2 K. Van Daele, D. Balalta, S. Hoekx, R. Jacops, N. Daems, T. Altantzis, D. Pant and T. Breugelmans, Synergy or Antagonism? Exploring the Interplay of SnO_2 and an N-OMC Carbon Capture Medium for the Electrochemical CO_2 Reduction toward Formate, *ACS Appl. Energy Mater.*, 2024, **7**, 5517–5527.
